# Use of Ultrasound-Guided Interfascial Plane Blocks in Anterior and Lateral Thoracic Wall Region as Safe Method for Patient Anesthesia and Analgesia: Review of Techniques and Approaches during COVID-19 Pandemic

**DOI:** 10.3390/ijerph19148696

**Published:** 2022-07-17

**Authors:** Marek Szamborski, Jarosław Janc, Joanna Rosińczuk, Jędrzej Jerzy Janc, Patrycja Leśnik, Lidia Łysenko

**Affiliations:** 1 Department of Anesthesiology and Intensive Therapy, 4th Military Clinical Hospital, 50-981 Wroclaw, Poland; marek.szamborski@gmail.com (M.S.); patrycja.lesnik@gmail.com (P.L.); 2 Department of Nursing and Obstetrics, Wroclaw Medical University, 51-618 Wroclaw, Poland; joanna.rosinczuk@umw.edu.pl; 3 Faculty of Medicine, Medical University of Lodz, 90-419 Lodz, Poland; jjjanc@o2.pl; 4 Department of Anesthesiology and Intensive Therapy, Wroclaw Medical University, 50-529 Wroclaw, Poland; lidia.lysenko@umw.edu.pl

**Keywords:** pectoral nerve plane block, serratus anterior plane block, transversus thoracic muscle plane block, parasternal intercostal nerve block, intercostal nerve block, pectoral interfascial plane block, pain, COVID-19, patient safety

## Abstract

Ultrasound-guided interfascial plane blocks performed on the anterior and lateral thoracic wall have become an important adjuvant method to general anesthesia and an independent method of local anesthesia and pain management. These procedures diminish the harmful effects of anesthesia on respiratory function and reduce the risk of phrenic nerve paralysis or iatrogenic pneumothorax. In postoperative pain management, interfascial plane blocks decrease the dosage of intravenous drugs, including opioids. They can also eliminate the complications associated with general anesthesia when used as the sole method of anesthesia for surgical procedures. The following procedures are classified as interfascial plane blocks of the anterior and lateral thoracic wall: pectoral nerve plane block (PECS), serratus anterior plane block (SAP), transversus thoracic muscle plane block (TTP), pectoral interfascial plane block (PIF), and intercostal nerve block (ICNB). These blocks are widely used in emergency medicine, oncologic surgery, general surgery, thoracic surgery, cardiac surgery, orthopedics, cardiology, nephrology, oncology, palliative medicine, and pain medicine. Regional blocks are effective for analgesic treatment, both as an anesthesia procedure for surgery on the anterior and lateral thoracic wall and as an analgesic therapy after trauma or other conditions that induce pain in this area. In the era of the COVID-19 pandemic, ultrasound-guided interfascial plane blocks are safe alternatives for anesthesia in patients with symptoms of respiratory distress related to SARS-CoV-2 and appear to reduce the risk of COVID-19 infection among medical personnel.

## 1. Introduction

With the development of interventional medicine and surgery, it is possible to carry out more complicated procedures in patients with a high burden of chronic diseases. Due to the growing group of patients taking medications influencing coagulation, including platelet aggregation, it is necessary to search for safe methods of anesthesia that have little effect on the circulatory or respiratory systems. In particular, in patients with cardiovascular disease and patients after COVID-19 infection, general anesthesia or central blockade may pose a risk of postoperative respiratory failure or may not be possible due to the use of anticoagulants. With the development of ultrasonography, it is possible to safely and fully control regional blockades, which can be an alternative to pain management in case of contraindications to taking nonsteroidal analgesics. The use of a regional blockade further reduces the risk of drug interactions.

Ultrasonography procedures in anesthesiology and intensive care have allowed the popularization of regional anesthesia as an effective treatment for acute and chronic pain. Regional anesthesia performed by a fascial plane block has become an important adjuvant method to general anesthesia, and an independent method of local anesthesia and pain management. In 2019–2021, the high risk of exposure associated with airway instrumentation during SARS-CoV-2 infection led to increased focus on regional anesthesia as an alternative to intubation and general anesthesia. The European Society of Regional Anaesthesia (ESRA), the American Society of Regional Anesthesia (ASRA), and *Pain Medicine* have published a common statement in their recommendations regarding the possibility of regional blocks as a safe method of patient anesthesia [1]. These groups also emphasized that regional blocks are safer for medical personnel and patients in the era of COVID-19 [2].

The choice of the regional anesthesia method should be guided by its negligible impact on respiratory function, reducing the risk of phrenic nerve palsy or iatrogenic pneumothorax. Furthermore, the doses administered to reduce the possibility of a toxic reaction after administering local anesthetics should minimize the risk of respiratory depression requiring oxygen therapy. As part of postoperative pain management, regional anesthesia also reduces the doses of intravenous analgesics used, particularly opioids.

Ultrasound-guided interfascial blocks of the anterior and lateral thoracic wall are safe and relatively easy to perform. Therefore, they are widely used in emergency medicine, oncologic surgery, general surgery, thoracic surgery, cardiac surgery, orthopedics, cardiology, nephrology, oncology, palliative medicine, and pain medicine [3,4].

This paper presents a systematized review of the regional fascial blocks used for procedures performed within the anterior and lateral thoracic wall. The principles of their selection are presented according to their method, intended effect, and location of the surgery. The techniques for performing particular blocks and their typical clinical applications are described. Finally, the importance of fascial blocks in anesthesiology and analgesic treatment is discussed, along with the current principles of regional blocks during the COVID-19 pandemic.

## 2. Local Thoracic Plain Blocks—Review of Techniques

**Figure 1:**
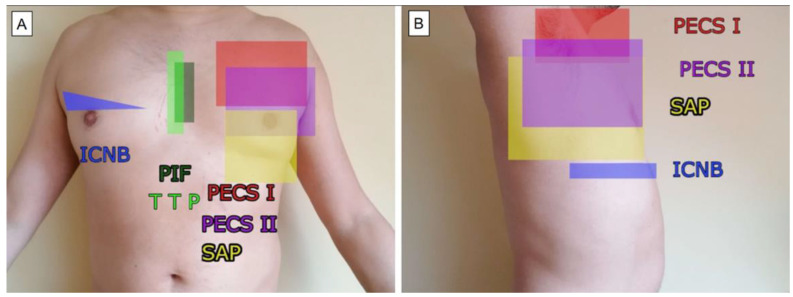
Approximate action areas for thoracic regional blocks: frontal (**A**) and lateral (**B**) views.

The regional blocks of the anterior and lateral thoracic wall include the pectoral nerve plane block (PECS), serratus anterior plane block (SAP), transversus thoracic muscle plane block (TTP), pectoral interfascial plane block (PIF), parasternal intercostal nerve block (PSI), and intercostal nerve block (ICNB) (Figure 1).

**Figure 2:**
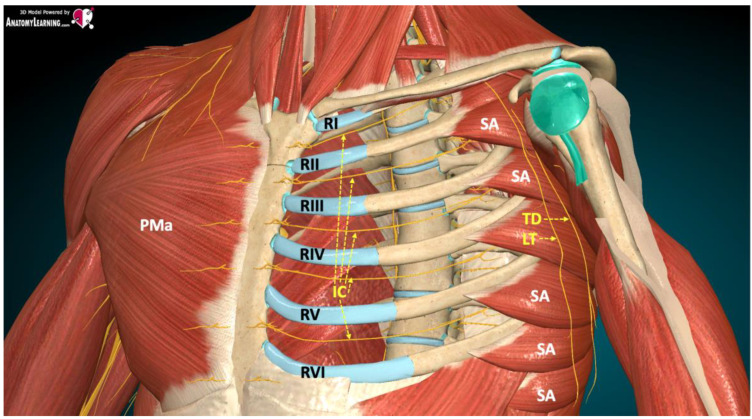
Neuroanatomy of the anterior and lateral thoracic wall [5]. Legend: RI, first rib; RII, second rib; RIII, third rib; RIV, fourth rib; RV, fifth rib; RVI, sixth rib; PMa, pectoralis major muscle; SA, serratus anterior muscle; IC, intercostal nerves; TD, thoracodorsal nerve; LT, long thoracic nerve.

The anterior and lateral thoracic wall is innervated by short branches of the brachial plexus, ventral branches of spinal nerves Th2–Th6 (intercostal nerves 2–6), and supraclavicular nerves (C3–C4), which are branches of the cervical plexus. The most important nerves in this group include the thoracic (medial and lateral), thoracodorsal, subscapular (upper and lower), axillary, long thoracic, and cutaneous intercostal nerves (lateral and anterior branches). The neuroanatomy of the anterior and lateral thoracic wall is shown in Figure 2.

### 2.1. Pectoral Nerve Block

**Figure 3:**
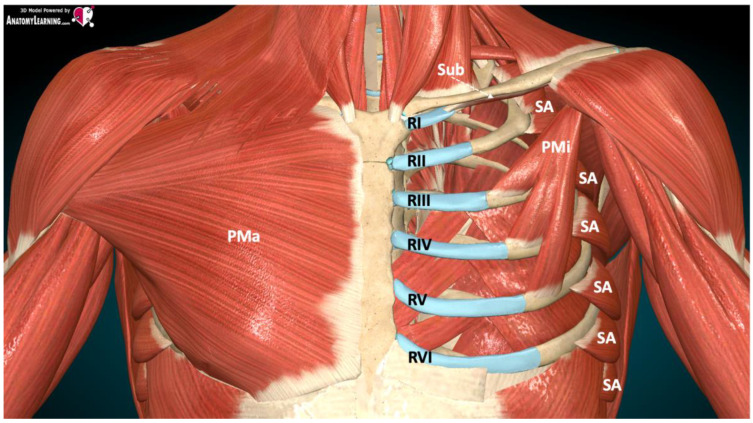
Muscle anatomy during the performance of PECS blocks [6]. Legend: RI, first rib; RII, second rib; RIII, third rib; RIV, fourth rib; RV, fifth rib; RVI, sixth rib; PMa, pectoralis major muscle; PMi, pectoralis minor muscle; SA, serratus anterior muscle; Sub, subclavian muscle.

Pectoral nerve block type I (PECS I) involves the medial and lateral thoracic nerves. This method was first described in 2011 by Blanco [6]. The anesthetic is deposited in the space between the pectoralis major and pectoralis minor muscles at the level of the third rib in the anterior axillary line, blocking the lateral and medial pectoral nerves (Figure 3). This provides anesthesia to the upper lateral quadrant of the thoracic wall. Initially, this method was dedicated to low-invasive breast procedures.

Pectoral nerve block type II (PECS II) is an improvement of the PECS I block, described by Blanco [7]. In addition to the medial and lateral thoracic nerves, PECS II includes the long thoracic nerve, thoraco-dorsal nerve, and lateral cutaneous branches of the intercostal nerves Th2–Th6. The anesthetic is deposited into two spaces: the space between the pectoralis minor and pectoralis major muscles, and the pectoralis minor and serratus anterior muscles at the level of the third rib in the midaxillary line. This provides anesthesia to the upper lateral quadrant of the thoracic wall and the axillary fossa region. The technique was originally used for painful reconstructive breast procedures and radical axillary lymph node resection anesthesia.

**Figure 4:**
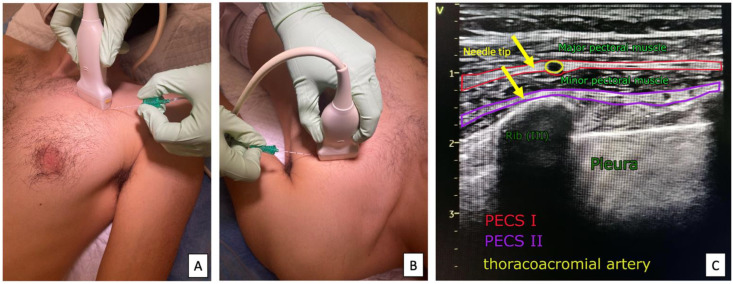
Ultrasound transducer positioning of the PECS I (**A**) and PECS II (**B**) blocks and sonoanatomy of the PECS blocks (**C**).

*Technique:* The classic approach to performing PECS I and PECS II blocks is to localize the anatomic structures using a linear ultrasound transducer (with a standard frequency range of 5–12 MHz). The patient is supine, with their arms along the body and the head slightly rotated in the direction opposite to the blockade performed. The head is placed parallel to the clavicle and in the midclavicular line. After locating the vessels in the midclavicular line below the clavicle, the ultrasound transducer is inferiorly and laterally moved until the third rib is visualized. The sonoanatomical image identifies the pectoralis major, pectoralis minor, and serratus muscles, with the fascial spaces between them. The characteristic sonoanatomical landmark is the thoracic branch of the thoracoacromial artery running in the space between the pectoral minor and major muscles. The medial distribution of anesthetic for PECS I blocks allows for anesthesia of the anterior cutaneous branches of the intercostal nerves; in turn, lateral distribution may involve the lateral cutaneous branches of the intercostal nerves. In PECS II, the medial distribution increases the extent of the lateral thoracic wall block mainly supplied by the long thoracic nerve and dorsal thoracic nerve, involving the axillary fossa, subclavian region, and deltoid muscle (Figure 4). The volume of LA used is shown in Table 1.

*Application:* The PECS I and PECS II blocks are used in a wide range of procedures, such as implantation of vascular catheters, intravenous ports, punctures, continuous drainage of pleural cavity (mainly PECS I); mastectomy; minimally invasive aortic valve replacement; thoracoscopy; minithoracotomy; rib fractures; postmastectomy pain syndrome; neoplastic pain; and pacemaker/cardioverter implantation (mainly PECS II) [5,8,9,10].

*Mini-review:* In recent years, there has been a significant spread of PECS blockade use. Its ease and universality of use have contributed to the expansion of available applications in thoracic surgery, e.g., in analgesic treatment after cardiac surgery of mitral/tricuspid valve replacement [11]. In addition, randomized trials are increasingly describing a potency comparable to that of paravertebral block (PVB), which is used for oncological and thorax surgery [12]. The safety of use associated with the limitation of general anesthesia drugs has opened up opportunities for using PECS II in obstetrics and gynecology [13]. An increasing number of patients with significant contraindications to general anesthesia have the opportunity to qualify for extensive vascular procedures using PECS II block [14]. The PECS II block is constantly modified to individual needs. Tuglar et al. [15] described a significant modification of the blockade called PECS-zero for breast surgery in an obese patient. Its new modifications result in greater comfort for the patient and the operator, being helpful for vascular access port implantation using cephalic vein venesection [16].

### 2.2. Serratus Anterior Plane Block

The Serratus anterior plane block (SAP) was developed in 2013 by Blanco et al. [17] during the analysis of adjacent structures at the PECS II block. This method significantly extends the possibility of analgesia in the lateral and inferior-lateral regions of the thoracic wall. SAP blocks includes the lateral cutaneous branches of intercostal nerves, located below the serratus anterior muscle within the region of Th3–Th9. The superficial variant of the block also includes the long thoracic and thoraco-dorsal nerve. Local anesthetics are administered in the fourth and fifth rib regions into the fascial space between the serratus anterior muscle and the latissimus dorsi muscle (superficial variant) or under the anterior alveolar muscle (deep variant). The needle insertion site is located between the midaxillary and posterior axillary lines.

**Figure 5:**
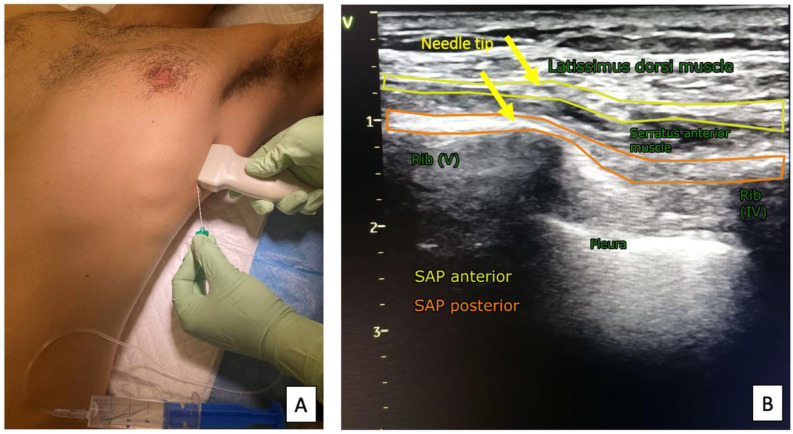
Ultrasound transducer positioning (**A**) and sonoanatomy of the SAP block (**B**).

*Technique:* The anatomical structures are localized using a linear ultrasound transducer (with a standard frequency range of 5–12 MHz). The patient is supine with an arm abducted at least 90° on the block side. After locating the vessels in the midclavicular line just below the clavicle, the ultrasound transducer is laterally moved and rotated by 90°, positioned along the vertical axis of the thorax to reach the lateral sectors of intercostal IV and V in the midaxillary line. When identifying the major and minor pectoralis muscles, ribs, with their neurovascular bundles, are moved over the latissimus dorsi and serratus anterior muscle. Directing the needle “on the rib” facilitates the correct deposition of local analgesics. Depending on the selected variant of the SAP anesthesia, the drug is administered above or below the serratus anterior muscle (Figure 5). The extent of anesthesia depends on the volume of anesthetic deposited [18]. The volume of LA used is shown in Table 1.

*Application:* The SAP block is used in mini-thoracotomies and classical thoracotomies; thoracoscopy; minimally invasive cardiac surgeries (aortic valve replacement); electrophysiological implantations, for example, subcutaneous implantable cardioverter-defibrillators (SICD); pain management after a rib injury; or implantation of tunneled permanent catheters [19,20,21,22,23].

*Mini-review:* The SAP block is often used in combined anesthesia in thorax and cardiac surgery [24,25]. In addition to providing significant hemodynamic stability and reduced opioid consumption, it also has a very good analgesic effect [26,27]. A novelty is using the SAP block as an element of analgesia for epigastric procedures [28,29].

### 2.3. Transversus Thoracic Muscle Plane Block

The transversus thoracic muscle plane block (TTP) involves depositing an anesthetic in the space between the transversus thoracis muscle and intercostal muscles. The extent of anesthesia corresponds to the medial part of the sternum, depending on which side of the block is performed, along with the medial part of the thoracic wall. The blockade includes the anterior ends of the intercostal nerve branches. The method was described in 2005 as a parasternal block and is performed without ultrasound guidance. This technique is used to reduce the requirement for analgesics after sternotomy following cardiac surgery [30]. In a TTP block, the nerve structures mentioned above in the range of Th2–Th6 are anesthetized [31].

**Figure 6:**
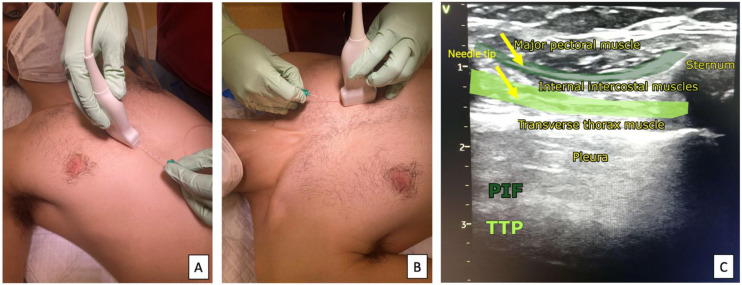
Ultrasound transducer positioning of the TTP (**A**) and PIF (**B**) blocks and sonoanatomy of the TTP and PIF blocks (**C**).

*Technique:* A linear ultrasound transducer (with a standard frequency range of 5–12 MHz) is placed parallel to the sternum in the parasternal line, sonoanatomically identifying the space between the second and fourth ribs. The needle is cranially directed, starting from the lower intercostal region. Due to the rich vascularization, injury to the venous perforators and intercostal veins, which run down the internal thoracic vein, should be avoided. The needle is successively passed through the pectoralis major and the external and internal intercostal muscles, reaching the surface of the transverse muscle, where the local anesthetic is deposited (Figure 6). Damage to the internal mammary artery, which gives off smaller branches in this area, should be avoided [31]. The closeness of the vascular structures, pleura, and pericardium makes this anesthesia challenging to achieve. The volume of LA used is shown in Table 1.

*Application:* The TTP block is especially useful in: sternotomy procedures; penetrating injuries of the sternal region; analgesic supplementation of the medial quadrants during breast surgery; subcutaneous implantation of cardiac defibrillators; and the treatment of pain syndromes, such as Tietze syndrome [32,33].

*Mini-review:* The transversus thoracis muscle is variable in many people and can be difficult to visualize with ultrasound. After coronary artery bypass grafting, patients can have tissue disruption in the transversus thoracis plane because of the internal mammary artery harvest, creating difficulties in transversus thoracis muscle identification. Subsequent reports have confirmed the high effectiveness of the block in the treatment of pain syndromes within the sternum region [34,35,36].

### 2.4. Pectoral Interfascial Plane Block

The pectoral interfascial plane (PIF) block is performed in the parasternal space between the pectoralis major and the internal intercostal muscles. The technique is very similar to the TTP block. The anesthesia covers the anterior ends of the intercostal nerve branches, relieving pain in the parasternal region and the sternal area on the block side [37,38].

*Technique:* Analogous to the TTP block, the linear ultrasound transducer (with a standard frequency range of 5–12 MHz) is positioned parallel to the sternum in the parasternal line, exposing the space between the pectoralis major and the intercostal muscles; anesthetic should be deposited in the layer between these muscles (Figure 6) [39]. The volume of LA used is shown in Table 1.

*Application:* The PIF block can be used to achieve analgesia after blunt chest trauma; in mechanical resuscitation; during breast and sternotomy procedures; in cases of Tietze syndrome [32,40].

*Mini-review:* PIF blocks are still mainly used in cardiac surgery and traumatology of sternal injuries. Due to the complicated technique and potential complications during its performance, the blockade technique has been modified, using safe anesthetics to perform it. Subsequent reports have confirmed the high effectiveness of the block in the treatment of pain syndromes within the sternum region [34,35,36].

### 2.5. Intercostal Nerve Block

The intercostal nerve block (ICNB) is most commonly performed as a pain management method and has little relevance in surgical procedures. This block involves targeted anesthetic delivery to the intercostal nerve region; the extent of anesthesia covers a specific intercostal space.

**Figure 7:**
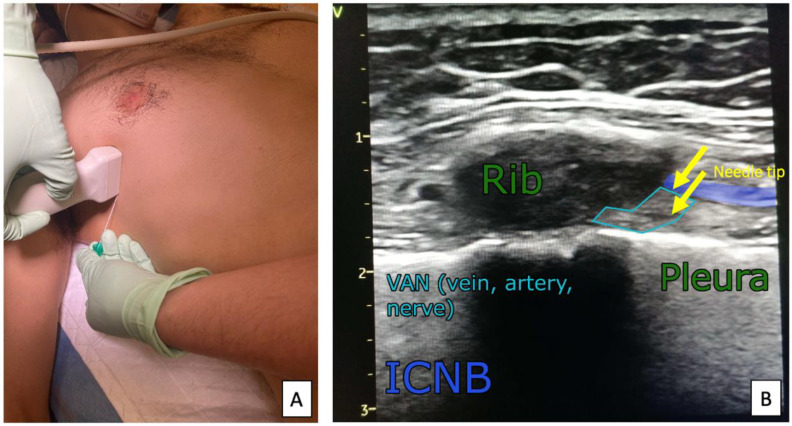
Ultrasound transducer positioning (**A**) and sonoanatomy of the ICNB block (**B**).

*Technique:* A linear ultrasound transducer (with a standard frequency range of 5–12 MHz) is placed in the near sagittal axis (parallel to the long axis of the rib), starting from the posterior axillary line (Figure 7). The intercostal space slightly differs in its content depending on the ultrasound transducer position—medial or lateral to the rib angle. Lateral to the rib angle, the vessels and nerves run between the internal intercostal muscle and the deepest groove of the upper rib. The vessels and nerves are located in the space between the internal intercostal membrane and the pleura, which is to the right of the rib angle. The analgesic is directly deposited over the pleura near the inferior margin of the rib (Figure 7). The volume of LA used is shown in Table 1.

*Application:* ICNB blocks can achieve adjuvant analgesia after traumatic rib fractures, and in thoracotomy; maintenance of pleural drainage; neuralgia caused, for example, by herpes zoster; cancer metastases; pain relief after extensive epigastric procedures; splenectomy; cholecystectomy; and nephrectomy [23,41].

*Mini-review:* ICNB, as a quick and simple procedure, is an alternative method to continuous epidural anesthesia in analgesic care [42]. In addition, the modification of the execution of the technique by changing the location of the anesthetic infiltration was described [15]. The analgesic effect of ICNB has also been observed in its application to urological treatments, which is quite surprising [43]. The safety of ICBN allows it to be used in patients for anesthesia in high-risk surgical procedures without the need for intubation [44].

## 3. Local Thoracic Plain Blocks—COVID-19 Approach

The importance of interfascial blocks in anesthesiology and pain management is widely appreciated [45]. Performing general anesthesia in patients with high surgical risk and multiple comorbidities significantly increases the risk of perioperative complications, prolongs the onset of rehabilitation in the postoperative period, and extends the length of hospital stay [46].

Interfascial anesthesia, instead of general anesthesia, can be used in patients with high perioperative risk for oncological surgery (breast surgery, lymph node dissection, and skin lesion removal); electrophysiological procedures (subcutaneous implantation of cardioverter-defibrillators or pacemaker devices); and pleural puncture or thoracoscopy. Furthermore, new local anesthetics, such as the left-handed enantiomers of bupivacaine or ropivacaine, can reduce the probability of symptoms of local anesthetic systemic toxicity (LAST). This improves the safety of postoperative care in patients after thoracic trauma or rib fractures [47]. The volume of LA used is shown in Table 1.

Ongoing studies of the anatomical structures of the chest wall are promising. A better understanding of these structures may help develop new regional anesthesia techniques, with the possibility of their application in minimally invasive surgeries, such as same-day surgery [48,49].

## 4. Interfascial Plane Blocks and the COVID-19 Approach

The COVID-19 pandemic affected the unfavorable perception of the use of general anesthesia concerning safety risks to operating room personnel. The use of general anesthesia in patients with respiratory infections due to SARS-CoV-2 significantly increases the risk of postoperative respiratory complications. The risk of respiratory depression in this group of patients is higher than in the general population. In the era of the COVID-19 pandemic, minimally invasive regional anesthesia techniques combined with an appropriate hygiene regimen appear to increase patient safety and reduce the risk of infection to operating room personnel [50,51,52].

Most of these techniques can serve as the only method of anesthesia and do not require additional sedation or general anesthesia. Techniques such as PECS, SAP, TTP, and PIF blocks can be used as the only method of anesthesia and postoperative pain management for selected procedures [6,7,8,16,53,54,55,56,57,58]. The intercostal nerve block is mainly used for pain management [38,42].

The COVID-19 pandemic has also restricted access to surgical procedures for uninfected patients and delayed treatment for patients with active infection. Patients with SARS-CoV-2 infection represent a group at increased perioperative risk, which correlates with both the effects of active SARS-CoV2 infection and the increasingly well-known and population-relevant long-term effects of COVID-19—the so-called ‘post-COVID-19 syndrome’ or ‘long COVID-19’—primarily in the respiratory and central nervous systems [59].

Analysis of the global COVID-19 statistics indicates no reasonable prognosis that the pandemic will end in the first half of 2022. Moreover, many European countries have a growing population of people vaccinated against COVID-19, mainly medical personnel, who must be prepared to provide services over the next few months to patients infected or recovering from COVID-19 [60,61]:

The following are the key recommendations for COVID-19 issued by ESRA/ASRA, dated 31 March 2020 [1]:

- Nonurgent procedures should be performed at a later date.
- Before regional anesthesia is administered, the extent and length of the procedure should be carefully considered.
- Any intraoperative conversions are highly undesirable; if the risk of conversion is high, it is better to perform general anesthesia from the beginning.
- Regional anesthesia, by definition, does not produce additional aerosol; however, if possible, it should be performed in an isolation room in COVID-19-positive patients; ultrasound-guided blocks should be performed.
- Regional anesthesia should be the method of choice in all cases without contraindications.

## 5. Conclusions

Considering the enormous development of surgical medicine and the popularization of ultrasonography, using regional blocks both in anesthesiology and for analgesia brings enormous benefits. Owing to the use of ultrasound, the risk of complications is minimal and the drugs act locally. They are often the only alternative to general anesthesia in patients using drugs that affect coagulation. Furthermore, they are an alternative to oral or intravenous analgesics in patients hospitalized in intensive care units. A reduced need for nonsteroidal anti-inflammatory drugs or opioids can be obtained owing to regional blockages.

Regional blocks are an effective method of analgesic treatment, both for anesthesia in surgical procedures on the anterior and lateral thoracic wall and for analgesic therapy after trauma or conditions inducing pain in this area. Ultrasound-guided techniques and modern, minimally toxic local anesthetics significantly increase patient safety and comfort. In the era of the COVID-19 pandemic, fascial block techniques provide a safe alternative to anesthesia for patients with symptoms of respiratory distress related to SARS-CoV-2. Moreover, these approaches appear to reduce the risk of infection of medical personnel.

## Figures and Tables

**Table 1:** Local anesthetic volumes used for described blocks.

Block Type	PECS I	PECS II	SAP	TTE	PIF	INCB
Suggested volume of local anesthetic (mL)	10	2 × 10	2 × 15	15	10	5
Range of volumes (mL)	10–15	10–20	20–30	5–20	4–12	4–10

Note: Type and concentration of LA depend on the preferences of the unit.

## Data Availability

Not applicable.
